# A clinical decision framework for redox-adapted, EMT-high cancers: From ferroptosis resistance to precision therapeutic stratification

**DOI:** 10.1016/j.redox.2026.104111

**Published:** 2026-03-04

**Authors:** Moon Nyeo Park, Hyo Jeong Kim, Sohyun Park, Rony Abdi Syahputra, Domenico V. Delfino, Seong-Gyu Ko, Bonglee Kim

**Affiliations:** a College of Korean Medicine, Kyung Hee University, 1-5 Hoegidong, Dongdaemun-gu, Seoul, 02447, Republic of Korea; b Department of Pharmacology, Faculty of Pharmacy, Universitas Sumatera Utara, Medan, Sumatera Utara, 20155, Indonesia; c Department of Medicine and Surgery, Piazza Università 1, Perugia, 06123, Italy; d Korean Medicine-Based Drug Repositioning Cancer Research Center, College of Korean Medicine, Kyung Hee University, Hoegi-dong Dongdaemun-gu, Seoul, 02447, Republic of Korea

**Keywords:** Redox adaptation, Ferroptosis resistance, NRF2 signaling, State-based oncology, Biomarker-guided stratification, Type 2 diabetes–associated cancer

## Abstract

Therapeutic resistance in advanced solid tumors increasingly reflects the emergence of adaptive tumor states rather than the absence of actionable molecular targets. Among these, redox adaptation represents a clinically decisive program that integrates ferroptosis resistance, epithelial–mesenchymal transition (EMT)–driven plasticity, metabolic rewiring, immune evasion, and extracellular vesicle–mediated communication. Here, we propose a state-based redox–EMT framework that explicitly translates these adaptive programs into clinical decision rules for patient stratification, therapeutic sequencing, and trial design. We synthesize mechanistic and translational evidence demonstrating that NRF2-centered antioxidant buffering and multilayered ferroptosis defense constitute an independent resistance axis that frequently supersedes oncogene dependency, particularly in metabolically compromised hosts such as patients with type 2 diabetes–associated pancreatic ductal adenocarcinoma (PDAC). Building on this concept, we delineate discrete redox–EMT tumor states, ranging from redox-low, EMT-restricted phenotypes to terminal redox-locked ecosystems characterized by ferroptosis resistance, stromal insulation, and immune exclusion. Importantly, we translate these states into actionable clinical decision rules, linking ferroptosis defense markers (e.g., GPX4, SLC7A11, FSP1), NRF2 activity, EMT status, and host metabolic context to rational selection and sequencing of cytotoxic therapy, targeted agents, redox modulation, and immunotherapy. We further outline a biomarker-guided stratification strategy integrating tissue-based and liquid biopsy readouts, including circulating tumor DNA, exosomal PD-L1, and redox-responsive microRNAs, to enable dynamic monitoring of tumor redox states during treatment. By reframing redox adaptation as a measurable, stratifiable, and targetable tumor state, this work provides a decision-oriented roadmap for state-aware precision oncology. Collectively, our framework supports a shift from mutation-centric treatment escalation toward clinical algorithms that anticipate and intercept redox-adapted therapeutic resistance, with direct implications for biomarker-enriched trials and adaptive treatment strategies in PDAC and beyond.

## Highlights

• Therapeutic resistance in PDAC reflects adaptive redox states reprogramming rather than target absence
• Redox remodeling integrates ferroptosis resistance, EMT plasticity, metabolic stress, and immune evasion.
• NRF2-centered buffering sets ferroptosis thresholds in EMT-high tumors.
• Redox state– stratification enables decision logic beyond mutation-based models.
• Redox-guided models link tissue and liquid biomarkers to therapeutic sequencing.

## Graphical abstract

**Figure:**
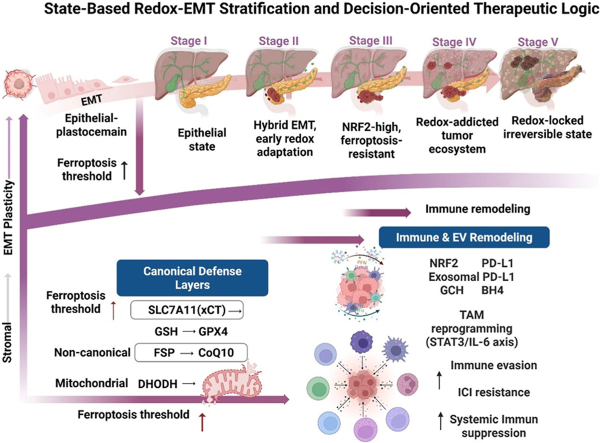

## 1 Introduction

### 1.1 Therapeutic failure as a consequence of host-conditioned redox-adapted tumor states

Cancer treatment has advanced substantially over recent decades; however, durable responses remain elusive in many solid tumors due to the emergence of adaptive resistance programs that transcend individual drug classes. Accumulating evidence indicates that redox adaptation constitutes a unifying biological framework through which cancer cells evade cytotoxic, targeted, and immune-based therapies, rather than a mere byproduct of treatment-induced stress. Persistent oxidative pressure within the tumor microenvironment (TME) selectively favors tumor subpopulations capable of rewiring antioxidant defenses, metabolic flux, and cell fate programs, thereby promoting therapeutic tolerance rather than vulnerability [1,2]. Importantly, redox adaptation does not reflect a uniform elevation of antioxidant capacity, but instead represents context- and compartment-specific optimization of reactive oxygen species (ROS) signaling thresholds, enabling tumor cell survival while preserving pro-tumorigenic signaling outputs [3]. A key consequence of this selective pressure is the emergence of drug-tolerant persisted cells and minimal residual disease (MRD), which are increasingly recognized as redox-buffered cellular reservoirs rather than genetically fixed resistant clones [4,5]. These persisted states exhibit reversible metabolic and transcriptional adaptations that allow survival under lethal therapeutic stress while retaining the capacity to re-enter proliferative programs upon treatment withdrawal [5]. A central component of redox-mediated therapeutic adaptation is epithelial–mesenchymal transition (EMT), a reversible phenotypic reprogramming in which epithelial cancer cells acquire mesenchymal, invasive, and stem-like properties. EMT is not merely a marker of tumor aggressiveness but functions as an active resistance state that attenuates oncogene dependency and suppresses drug-induced cell death signaling [1,6]. Recent single-cell and lineage-tracing studies further demonstrate that EMT-associated resistance is most pronounced in partial or hybrid EMT states, rather than fully mesenchymal extremes, underscoring phenotypic plasticity—rather than terminal differentiation—as the principal driver of therapy tolerance [7].

In pancreatic ductal adenocarcinoma (PDAC), for example, EMT-driven plasticity enables tumor cells to bypass KRAS dependency through TGF-β–NFAT5–SMAD3/4 signaling, thereby rendering KRAS-targeted therapies transiently effective at best [1]. Notably, this apparent loss of KRAS dependency does not represent oncogenic disengagement but rather a state transition in which KRAS-driven redox programs are re-routed through NRF2-centered antioxidant circuitry. In KRAS-mutant PDAC, both canonical and non-canonical NRF2 activation—mediated by p62 accumulation, GSK3β inhibition, and β-TrCP interference—sustain SLC7A11–GSH–GPX4–dependent ferroptosis defense even under pharmacologic KRAS suppression [[8], [9], [10]]. Consistently, oncogenic KRAS signaling itself promotes redox adaptation via NRF2-dependent antioxidant programs, either through metabolic ROS accumulation or suppression of KEAP1-mediated NRF2 degradation, particularly in PDAC models [11,12]. This bypass phenomenon exemplifies a broader principle whereby redox-adapted EMT states decouple tumor survival from canonical oncogenic drivers, thereby limiting the durability of pathway-selective inhibitors across tumor types [13]. Importantly, EMT is tightly coupled to redox remodeling, such that mesenchymal-like cancer cells exhibit enhanced antioxidant capacity, altered mitochondrial dynamics, and increased reliance on glutathione- and thioredoxin-based buffering systems, collectively allowing survival under sustained oxidative pressure [14].

Mechanistically, EMT induction in KRAS-mutant PDAC is accompanied by selective enrichment of NRF2-high, ferroptosis-resistant subclones, indicating that EMT functions as a redox-buffering state rather than merely a migratory phenotype [15,16]. This transition enables tumor cells to survive KRAS pathway inhibition by substituting oncogene addiction with antioxidant dependency, effectively uncoupling survival from KRAS signaling intensity [17,18]. In KRAS-mutant epithelial tumors, EMT induction has been shown to coincide with sustained NRF2 activation, leading to transcriptional upregulation of SLC7A11, GPX4, and other ferroptosis-suppressive genes even under pharmacologic KRAS inhibition [19,20]. At the molecular level, this remodeling involves coordinated NRF2-driven transcriptional reprogramming, suppression of lipid peroxidation cascades, and stabilization of mitochondrial redox homeostasis, collectively raising the threshold for therapy-induced oxidative catastrophe [21]. Consequently, EMT-associated redox adaptation directly converges with ferroptosis resistance, a central mechanism underlying failure of both conventional chemotherapy and emerging redox-targeted strategies. Although ferroptosis represents a major vulnerability in therapy-resistant tumors, EMT-high and stem-like cancer cells frequently suppress ferroptotic execution through GPX4, SLC7A11, and NRF2-mediated antioxidant programs [14,22]. KRAS-driven metabolic rewiring further reinforces this ferroptosis-resistant phenotype by enhancing glutamine flux, NADPH generation, and NRF2 transcriptional output, particularly within EMT-high and stem-like compartments [23,24]. As a result, KRAS inhibition alone fails to destabilize lipid peroxide control, explaining why KRAS-targeted therapies often induce only transient tumor regression prior to redox-adapted relapse [25,26]. Indeed, KRAS-driven NRF2 activation has emerged as a central upstream regulator of ferroptosis resistance in PDAC, functionally insulating tumor cells from lipid peroxidation even when KRAS–MAPK signaling is partially suppressed [27,28].

Importantly, ferroptosis resistance in redox-adapted tumors is dynamic rather than static, suggesting that temporal disruption of redox buffering—rather than constitutive pathway blockade—may be required for effective therapeutic exploitation [29]. Beyond intrinsic tumor cell survival, redox-adapted EMT states actively remodel the immune microenvironment. EMT-associated tumors exhibit elevated secretion of immunosuppressive cytokines, including interleukin-6 (IL-6), which attenuate cytotoxic T cell and natural killer cell activity while promoting regulatory T cell and Th17 polarization [30]. Cooperative KRAS–NRF2–IL-6 signaling axes further establish immune-excluded tumor microenvironments, reducing immune-mediated oxidative pressure and stabilizing redox-tolerant EMT states [31,32]. Beyond its canonical role in antioxidant defense, NRF2 activation functionally intersects with immune evasion programs in cancer stem–like cells. NRF2-driven stabilization of SLC7A11–GPX4–dependent ferroptosis resistance is accompanied by sustained PD-L1 expression and attenuation of ROS-dependent immunogenic signaling. This coordinated redox–immune coupling establishes a permissive niche in which CSCs evade both ferroptotic collapse and immune-mediated elimination, thereby contributing to durable resistance under metabolic and therapeutic stress [33,34].

This cytokine-driven immune rewiring creates a permissive niche for therapy-resistant cell persistence by suppressing immune-mediated oxidative stress [35]. In EGFR-mutant non-small cell lung cancer (NSCLC), EMT-driven IL-6 signaling has been shown to directly impair responsiveness to immune checkpoint blockade, explaining the poor efficacy of PD-1/PD-L1 inhibitors in TKI-resistant settings [30,36]. Collectively, these findings indicate that therapeutic failure in advanced cancers cannot be adequately explained by genetic alterations alone. Instead, redox-adapted EMT states function as dynamic resistance hubs that integrate ferroptosis evasion, metabolic rewiring, immune suppression, and lineage plasticity into a coordinated survival architecture. This evidence supports a state-based redox oncology paradigm, in which oncogenic drivers such as KRAS remain biologically active but are rendered therapeutically subordinate to NRF2-centered redox survival circuits [37]. Accordingly, KRAS should be viewed not as an isolated oncogenic dependency but as an upstream amplifier of redox-adaptive circuitry, whose effective therapeutic targeting requires simultaneous disruption of NRF2-driven ferroptosis defense networks to achieve durable responses in PDAC [38]. This convergence necessitates a shift from mutation-centric resistance models toward state-based redox oncology, in which therapeutic success depends on destabilizing adaptive redox circuits rather than inhibiting single oncogenic nodes [39].

Finally, single-pathway inhibition remains insufficient unless adaptive redox–EMT circuits are disrupted at both cellular and microenvironmental levels. In metabolically dysregulated hosts, particularly those with type 2 diabetes, chronic hyperinsulinemia and sustained insulin/IGF-1 receptor signaling generate persistent mitochondrial and cytosolic ROS, which paradoxically stabilize NRF2 activity rather than induce oxidative collapse [11,12]. In T2DM-associated PDAC, redox adaptation should be understood as a host-imprinted trait rather than a purely tumor-acquired resistance mechanism, with chronic systemic oxidative and metabolic stress preconditioning both tumor cells and their microenvironment prior to therapeutic exposure. This endocrine-driven redox pressure preconditions tumor ecosystems toward antioxidant dependency, selectively favoring survival and expansion of NRF2-high, ferroptosis-resistant subpopulations within EMT-high and stem-like cancer compartments [19,40].

Activated pancreatic stellate cells act as metabolic and inflammatory amplifiers within the tumor microenvironment by secreting IL-6 and CXCL12, thereby enforcing STAT3-dependent transcriptional reprogramming in cancer stem–like cells. This paracrine signaling axis stabilizes stemness programs while simultaneously enhancing ferroptosis resistance through coordinated activation of NRF2–SLC7A11–GPX4 antioxidant circuitry. Notably, PSC-derived extracellular vesicles deliver redox-modulatory cargos that further reinforce cystine–glutathione flux and lipid peroxide detoxification, functionally insulating CSCs from iron-dependent cell death [33,34,41,42].

Beyond tumor-intrinsic mechanisms, mesenchymal stem cell–like stromal populations—including pancreatic stellate cells and cancer-associated fibroblasts—reinforce redox adaptation through endocrine-like paracrine signaling. Under diabetic and inflammatory conditions, these stromal cells secrete IL-6, TGF-β, and redox-modulatory extracellular vesicles that enhance NRF2 activation, glutathione buffering, and lipid peroxide detoxification in neighboring cancer stem–like cells, thereby stabilizing ferroptosis resistance within the tumor niche [[43], [44], [45]]. Collectively, these observations support a functional convergence in which EMT-high cancer stem–like cells operate as redox-primed stem states, phenotypically and metabolically resembling mesenchymal stromal cells intrinsically adapted to oxidative and metabolic stress [46,47]. This convergence provides a mechanistic explanation for the heightened therapeutic resistance observed in metabolically compromised patients. Unlike prior reviews that frame redox signaling as a downstream consequence of oncogenic or metabolic stress, this work positions redox adaptation as a primary state variable that governs therapeutic decision-making across EMT, ferroptosis, and immune axes.

## 2 EMT-associated therapy resistance

Redox adaptation in cancer should no longer be regarded as an abstract theoretical construct but instead as a clinically observable and functionally stratifiable tumor state that directly governs therapeutic vulnerability [48]. Accumulating evidence demonstrates that EMT is not a binary switch but comprises a continuum of discrete tumor cell states, including partial and hybrid epithelial/mesenchymal phenotypes, each endowed with distinct functional properties relevant to therapy resistance [49,50]. Single-cell transcriptomic and lineage-tracing studies now provide direct experimental evidence that these intermediate EMT states represent stable yet reversible cellular programs governed by distinct gene regulatory networks (GRNs), rather than transient intermediates or simple mixtures of epithelial and mesenchymal populations [51,52]. Importantly, tumor cells residing in intermediate or hybrid EMT states exhibit the highest degree of phenotypic plasticity, enhanced tumor-initiating capacity, and pronounced resistance to apoptosis, rendering them disproportionately enriched following chemotherapy, targeted therapy, and immunotherapy [53]. Dynamic EMT/MET switching has been visualized in live tumor cells using dual fluorescent sensor systems, demonstrating that therapeutic stress actively selects for EMT-plastic subpopulations capable of oscillating between epithelial and mesenchymal states [54,55]. These EMT-high populations frequently overlap with cancer stem cell–like compartments and persist as minimal residual disease, thereby providing a cellular reservoir for relapse and metastatic progression [56,57]. Notably, EMT-associated drug-tolerant persisted cells often exhibit slow-cycling or dormant phenotypes that enable survival during therapeutic bottlenecks while retaining the capacity to re-enter proliferative programs upon treatment withdrawal, directly linking EMT plasticity to the biology of minimal residual disease [58,59].

**Fig. 1:**
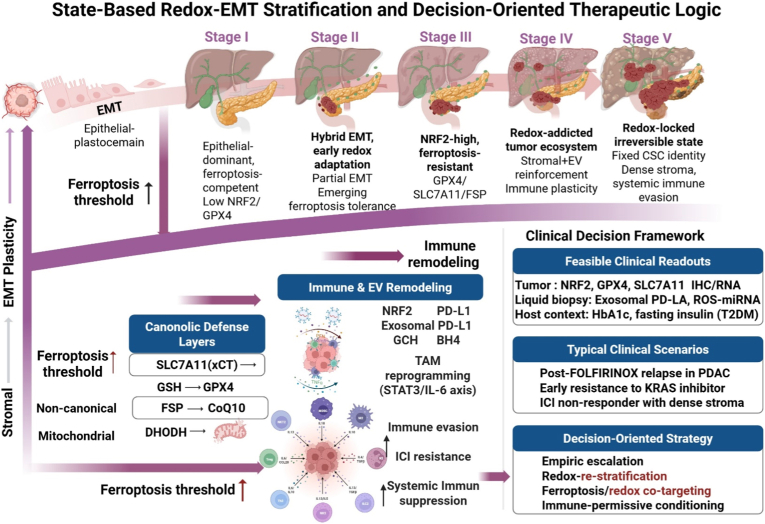
State-based redox–EMT framework linking ferroptosis defense and immune evasion in diabetes-associated PDAC. Schematic illustration of discrete tumor states along the EMT continuum, highlighting progressive redox adaptation, elevation of the ferroptosis threshold, and coordinated immune remodeling. The framework integrates metabolic stress, stromal–tumor interactions, multilayered ferroptosis defense mechanisms, and clinically actionable biomarkers to support decision-oriented therapeutic stratification in PDAC. Abbreviations: ↑, upregulation; ↓, downregulation; Advanced glycation end products (AGEs); B tetrahydrobiopterin (BH4); Cancer stem cell (CSC); Chemokine (*C*-X-C motif) ligand 12 (CXCL12); Dihydroorotate dehydrogenase (DHODH); Epithelial–mesenchymal transition (EMT); Extracellular vesicle (EV); Ferroptosis suppressor protein 1 (FSP1); Glutathione (GSH); Glutathione peroxidase 4 (GPX4); GTP cyclohydrolase 1 (GCH1); Interleukin-6 (IL-6); Immune checkpoint inhibitor (ICI); Kelch-like ECH-associated protein 1 (KEAP1); Nuclear factor erythroid 2–related factor 2 (NRF2); Pancreatic ductal adenocarcinoma (PDAC); Reactive oxygen species (ROS); Solute carrier family 7 member 11 (SLC7A11); Tumor-associated macrophage (TAM); Transforming growth factor-β (TGF-β); Type 2 diabetes mellitus (T2DM).

Beyond transcriptional reprogramming, EMT is tightly coupled to metabolic and redox remodeling that enables cancer cells to withstand oxidative and therapeutic stress. EMT-associated cells display coordinated activation of antioxidant defense systems, including NRF2-driven transcriptional programs, cystine–glutamate antiporter SLC7A11 upregulation, and GPX4-mediated lipid peroxide detoxification, collectively conferring resistance to ferroptosis and ROS-induced cell death [60]. Metabolic rewiring during EMT further reinforces redox resilience through enhanced NADPH generation, altered mitochondrial dynamics, and suppression of lipid peroxidation, thereby functionally insulating EMT-high cells from ferroptotic collapse [61,62]. This convergence of phenotypic plasticity and redox resilience establishes a therapy-recalcitrant EMT–redox axis that has been consistently observed across solid and hematologic malignancies [63]. Cross-tumor single-cell atlases further reveal that EMT–redox programs recur as conserved cancer cell states across patients and histologies, largely independent of dominant oncogenic mutations, underscoring their role as pan-cancer resistance modules rather than disease-specific anomalies [52,64]. From a clinical standpoint, these findings challenge traditional histopathologic and mutation-centric classification systems. Instead, they support a state-based oncology framework in which tumors are stratified according to functional redox adaptation and EMT status. Integrating biomarkers that capture EMT spectrum positioning together with redox defense activation—such as hybrid EMT signatures, NRF2 pathway activity, and ferroptosis resistance markers—may substantially improve prediction of therapeutic response and enable rational patient stratification for redox- or EMT-targeted combination strategies [65,66]. Such state-based classification paradigms align with emerging precision oncology efforts that prioritize cellular behavior over static genotype, providing a conceptual foundation for adaptive, decision-oriented therapeutic strategies that anticipate rather than react to therapy-induced resistance [67,68]. As illustrated in Fig. 1, this framework conceptualizes tumor evolution in diabetes-associated PDAC as a progression across five functionally distinct redox–EMT states, rather than as a linear accumulation of genetic alterations. Early epithelial-dominant tumors remain ferroptosis-competent with low antioxidant buffering capacity, whereas transitional hybrid EMT states acquire partial redox tolerance through emerging NRF2 activation and metabolic rewiring. With further progression, tumors enter NRF2-high, ferroptosis-resistant states characterized by coordinated activation of canonical and non-canonical defense layers, including SLC7A11–GPX4, FSP1–CoQ10, and mitochondrial DHODH-dependent pathways. These redox-addicted states are reinforced by stromal signaling and extracellular vesicle–mediated immune remodeling, culminating in immune-excluded, therapy-refractory tumor ecosystems. Importantly, this state-based architecture provides a clinically actionable framework in which therapeutic resistance reflects elevated ferroptosis thresholds and immune suppression rather than failure of target inhibition per se, thereby supporting redox-guided re-stratification and rational combination strategies. In this framework, redox adaptation is operationalized as a set of discrete, clinically interpretable tumor states distributed along the EMT continuum, each directly linked to feasible tissue- and liquid-biopsy biomarkers and decision-oriented therapeutic logic. Notably, in T2DM-associated PDAC, chronic systemic oxidative and metabolic stress functions as an upstream conditioning force that accelerates progression toward NRF2-high, ferroptosis-resistant states even before therapeutic exposure (Fig. 1).

## 3 Mechanistic convergence underlying redox adaptation

At the core of redox-adapted tumor survival lies a multilayered ferroptosis defense architecture that operates across spatially distinct subcellular compartments. Canonical cytosolic protection is mediated by the System Xc^−^–glutathione (GSH)–glutathione peroxidase 4 (GPX4) axis, where upregulation of SLC7A11 ensures sustained cystine import and GSH biosynthesis, thereby neutralizing lipid hydroperoxides and suppressing ferroptotic execution [69,70]. Clinical transcriptomic analyses consistently associate elevated SLC7A11 and GPX4 expression with poor prognosis and therapeutic refractoriness across multiple solid tumors, underscoring ferroptosis evasion as a clinically relevant resistance phenotype [69]. Rather than functioning as a binary on/off switch for ferroptosis, the System Xc^−^–GPX4 axis establishes a quantitative buffering threshold that determines whether lipid peroxidation remains sublethal or propagates into irreversible membrane damage, thereby shaping therapy tolerance rather than absolute resistance [71]. However, ferroptosis resistance in advanced cancers is not exclusively dependent on GPX4 activity. Parallel antioxidant systems have been identified that compensate for GPX4 inhibition and confer robustness to oxidative stress. Ferroptosis suppressor protein 1 (FSP1, formerly AIFM2) acts independently of GPX4 by catalyzing NAD(P)H-dependent reduction of coenzyme Q10 (CoQ10) to its radical-trapping form, ubiquinol, at the plasma membrane [72,73]. This FSP1–CoQ10 axis establishes an alternative lipid peroxide detoxification route, providing a mechanistic basis for the limited efficacy of GPX4-targeted strategies in certain tumor contexts. Recent evidence further indicates that FSP1 activity is dynamically regulated by cellular redox demand and membrane lipid composition, enabling tumors to shift ferroptosis defense reliance between GPX4-dominant and FSP1-dominant modes under therapeutic pressure [74,75]. More recently, mitochondrial redox homeostasis has emerged as a third, spatially segregated layer of ferroptosis defense.

Dihydroorotate dehydrogenase (DHODH), localized to the inner mitochondrial membrane, suppresses mitochondrial lipid peroxidation by regenerating reduced CoQH_2_ within the mitochondrial compartment, independently of both GPX4 and FSP1 [70,76]. This compartmentalized protection is particularly relevant in tumors undergoing mitochondrial metabolic rewiring, in which ferroptotic signaling is initiated but fails to propagate due to preserved mitochondrial antioxidant buffering. Beyond antioxidant detoxification, ferroptosis sensitivity is fundamentally shaped by lipid remodeling programs that determine the availability of peroxidation-prone phospholipid substrates. Under chronic metabolic stress, activation of AMPK suppresses polyunsaturated fatty acid (PUFA) synthesis, thereby limiting PUFA-phospholipid incorporation into cellular membranes and reducing intrinsic susceptibility to ferroptotic damage. Concurrently, mechanotransduction pathways such as YAP/TAZ signaling transcriptionally regulate lipid remodeling enzymes and cystine transport systems, reinforcing a membrane composition that favors monounsaturated fatty acid (MUFA) enrichment and ferroptosis evasion. This dynamic reprogramming of membrane lipid architecture establishes a pre-peroxidation checkpoint that operates upstream of canonical GPX4-dependent detoxification, enabling stress-adapted tumor cells to tolerate lipid oxidative insults without engaging lethal ferroptotic cascades [77,78].

In parallel with lipid substrate availability, iron mobilization critically governs whether lipid peroxidation propagates into irreversible ferroptotic execution. Ferritinophagy, mediated by the cargo receptor NCOA4, selectively releases ferritin-bound ferrous iron, expanding the labile iron pool and catalyzing lipid radical amplification through Fenton chemistry. In stress-adapted tumor cells, tight regulation of ferritinophagy establishes an iron-buffering threshold that permits metabolic flexibility while preventing uncontrolled ferroptotic collapse. Pharmacological or phytochemical perturbation of this axis—such as NCOA4 activation—can destabilize iron homeostasis, thereby converting sublethal lipid peroxidation into cytotoxic ferroptotic signaling. This iron-dependent checkpoint integrates with lipid remodeling and antioxidant defenses to determine whether ferroptotic stress remains buffered or can be converted into a therapeutically exploitable vulnerability [[79], [80], [81]]. Importantly, DHODH-mediated ferroptosis defense links nucleotide metabolism with mitochondrial redox control, creating a metabolic–oxidative checkpoint that selectively sustains survival in GPX4-low or cystine-limited tumor states [67,75]. This mitochondrial safeguard introduces a spatial hierarchy in ferroptosis regulation, allowing lipid peroxidation to proceed in non-mitochondrial membranes while preserving mitochondrial integrity, thereby maintaining ATP production and stress adaptability [82].

Upstream of these ferroptosis defense modules, the NRF2–KEAP1 signaling axis functions as a master transcriptional coordinator of redox adaptation. Constitutive NRF2 activation—frequently driven by KEAP1 loss or oxidative stress selection—induces broad expression of SLC7A11, GPX4, FSP1-associated pathways, and iron-handling genes, collectively elevating the ferroptosis threshold [83]. Rather than acting as a linear antioxidant switch, NRF2 establishes a permissive transcriptional landscape that synchronizes ferroptosis defense with metabolic rewiring, proteostasis, and mitochondrial fitness, thereby stabilizing redox-adapted phenotypes under chronic therapeutic stress, while creating a permissive landscape for additional stress-buffering modules beyond direct antioxidant control [84,85].

In parallel with ferroptosis defense systems, chronic endoplasmic reticulum (ER) stress signaling emerges as a critical convergent mechanism sustaining redox-adapted tumor survival under prolonged metabolic and inflammatory pressure. Persistent activation of the protein kinase RNA-like ER kinase (PERK)–eukaryotic initiation factor 2α (eIF2α) axis enforces a translational reprogramming state that selectively attenuates global protein synthesis while uncoupling C/EBP homologous protein (CHOP)-dependent apoptotic execution. This adaptive ER stress program preserves cellular viability despite sustained proteotoxic and oxidative stress, particularly in cancer stem–like subpopulations exposed to chronic therapeutic challenge. Importantly, PERK–eIF2α–mediated stress tolerance functionally converges with ferroptosis-resistant programs, positioning ER stress adaptation as a bifurcation node through which tumor cells evade both apoptotic and iron-dependent cell death pathways. Such convergence further reinforces the stability of redox-adapted phenotypes by coordinating proteostasis, mitochondrial fitness, and lipid peroxidation control within a unified stress-buffering architecture [[86], [87], [88]].

Beyond metabolic and ferroptotic buffering, redox adaptation becomes epigenetically stabilized through DNMT1-dependent chromatin remodeling. Sustained oxidative and metabolic stress selectively reinforces DNA methylation programs that restrict transcriptional plasticity while preserving antioxidant and survival gene expression. Rather than representing a transient stress response, this DNMT1-driven epigenetic rigidity converts redox adaptation into a heritable cellular state, effectively encoding a form of stress memory. Such epigenetically fixed phenotypes enable tumor cells to rapidly reconstitute therapy-resistant programs upon renewed therapeutic pressure, even following periods of drug withdrawal. This coupling between redox stress and chromatin accessibility provides a mechanistic basis for durable therapy resistance and disease recurrence in metabolically conditioned cancers [89,90].

Beyond redox detoxification, NRF2 signaling intersects with metabolic reprogramming and immune evasion, thereby linking oxidative resilience to broader therapeutic resistance phenotypes. Redox adaptation further converges with adhesion signaling and extracellular vesicle (EV) remodeling, reinforcing phenotypic plasticity and stress tolerance. EMT-associated reorganization of focal adhesion signaling—often mediated through ROS-responsive Src–FAK pathways—facilitates cytoskeletal remodeling, migratory capacity, and survival under oxidative stress [91]. These adhesion changes are not purely structural but actively feed back into redox signaling by modulating intracellular ROS distribution and stress tolerance. ROS-sensitive adhesion complexes function as mechanochemical transducers, translating oxidative cues into sustained mesenchymal signaling states that reinforce ferroptosis resistance and therapy tolerance [68,92]. Concurrently, EV and exosome remodeling enables systemic propagation of redox-adaptive states. Tumor-derived exosomes enriched in immune-modulatory cargo, including PD-L1, redox-regulated microRNAs, and antioxidant enzymes, contribute to immune suppression and niche conditioning in distant microenvironments [93,94]. Such EV-mediated signaling effectively externalizes redox adaptation, allowing resistant tumor clones to influence surrounding stromal and immune compartments. Selective exosomal cargo loading is itself regulated by oxidative stress–responsive RNA-binding proteins and lipid peroxidation status, enabling redox-adapted cells to transmit ferroptosis-resistant and immune-evasive programs beyond the primary tumor site [2,95]. Collectively, these findings support a unifying model in which ferroptosis defense, NRF2-driven transcriptional control, adhesion signaling plasticity, and EV remodeling operate as an interconnected resistance network rather than isolated pathways. This network architecture explains why single-node redox interventions frequently yield only transient responses and underscores the necessity of combinatorial strategies that simultaneously disrupt cytosolic, mitochondrial, and extracellular layers of redox adaptation.

### 3.1 Temporal evolution of redox adaptation during tumor progression

Redox adaptation is not an instantaneous resistance phenotype but a temporally orchestrated process that evolves in parallel with tumor progression, therapeutic exposure, and microenvironmental remodeling. Longitudinal single-cell and lineage-tracing studies now demonstrate that cancer cells transition through discrete redox states rather than undergoing abrupt acquisition of antioxidant dominance [96,97]. Early-stage tumors typically retain a redox-vulnerable phenotype characterized by limited antioxidant buffering and preserved susceptibility to oxidative cell death. However, sustained metabolic stress, oncogenic signaling, and therapy-induced oxidative pressure progressively select for subpopulations capable of dynamically recalibrating ROS thresholds, thereby initiating redox adaptation as an adaptive survival program rather than a fixed trait [98,99].

During initial tumor expansion, transient activation of stress-response pathways—including NRF2, ATF4, and HIF-1α—functions as a reversible buffering mechanism that permits survival under fluctuating oxidative conditions [97,100]. At this stage, redox adaptation remains plastic and context-dependent, allowing tumor cells to oscillate between oxidative vulnerability and antioxidant engagement. Importantly, this early adaptive phase is tightly coupled to partial epithelial–mesenchymal transition (EMT) states, in which cells acquire metabolic flexibility and stress tolerance without committing to irreversible mesenchymal identity [99,101]. These intermediate states exhibit heightened redox sensitivity, enabling rapid rewiring of antioxidant systems in response to microenvironmental cues.

As tumors progress or are exposed to repeated therapeutic insults, redox adaptation undergoes stabilization through reinforcement of ferroptosis defense circuitry. Sustained activation of the NRF2–SLC7A11–GPX4 axis, together with compensatory engagement of parallel antioxidant modules such as FSP1–CoQ and mitochondrial DHODH pathways, progressively elevates the ferroptosis threshold [100,102]. This transition marks a critical inflection point at which redox adaptation shifts from inducible stress response to semi-fixed survival architecture. At this stage, tumor cells no longer merely tolerate oxidative stress but become functionally dependent on antioxidant buffering for survival, rendering redox homeostasis a dominant determinant of therapeutic response [102,103].

Temporal analyses further reveal that redox adaptation is reinforced by progressive microenvironmental co-evolution. Stromal fibroblasts, immune-suppressive myeloid populations, and extracellular vesicle–mediated signaling increasingly participate in maintaining antioxidant dominance by supplying cystine, glutathione precursors, cytokines, and redox-regulatory microRNAs [101,104]. These non–cell-autonomous inputs convert redox adaptation from a tumor-intrinsic phenomenon into an ecosystem-level property, thereby stabilizing therapy-resistant states even in the absence of continued drug pressure.

In advanced disease stages, redox adaptation becomes tightly interwoven with lineage plasticity, immune evasion, and metabolic rewiring, producing a redox-addicted tumor state in which oxidative stress is no longer cytotoxic but instead functions as a signaling currency that sustains malignancy [103,105]. Importantly, evidence suggests that once this late-stage redox-locked state is established, therapeutic attempts to induce oxidative collapse often fail due to rapid activation of compensatory antioxidant networks. This temporal hierarchy underscores that the clinical efficacy of redox-targeted interventions is highly contingent on timing, with maximal vulnerability occurring during intermediate adaptive windows rather than terminal antioxidant fixation [105,106].

Collectively, these observations support a temporal model of redox adaptation in which tumor progression is accompanied by stepwise reinforcement of antioxidant defenses, transitioning from reversible stress tolerance to entrenched ferroptosis resistance. Recognizing redox adaptation as a time-dependent process rather than a static phenotype provides a mechanistic basis for stage-specific therapeutic strategies and emphasizes the necessity of early intervention before redox addiction becomes irreversible [98,106].

## 4 Section 4. decision-oriented therapeutic strategies: redox State–Guided application of phytomedicine

Therapeutic resistance in advanced cancers increasingly reflects failure to match intervention strategies to tumor redox states rather than intrinsic inefficacy of individual agents. In this context, phytomedicine—characterized by multi-component, pleiotropic bioactivity—can be rationally repositioned not as a nonspecific adjunct, but as a redox state–modulating therapeutic modality. Emerging evidence suggests that the efficacy of phytochemical interventions is highly contingent upon the temporal and functional redox state of the tumor ecosystem, particularly along the EMT–ferroptosis–immune axis [107,108].

### 4.1 Phytomedicine in early redox-low/EMT-low states (state I–II)

In epithelial-dominant tumors with limited antioxidant buffering capacity and intact ferroptosis susceptibility, excessive induction of oxidative stress may be unnecessary and potentially counterproductive. In these early redox-low states, phytomedicine may function optimally as a redox-stabilizing and differentiation-preserving agent, attenuating metabolic stress signaling while preventing premature activation of adaptive antioxidant programs [109]. Several plant-derived compounds have been shown to modulate mitochondrial ROS production, glucose flux, and inflammatory signaling without triggering NRF2 overactivation, thereby maintaining ferroptosis competence and epithelial integrity [110]. From a decision-oriented perspective, phytomedicine in this context is best deployed preventively or maintenance-oriented, aiming to delay redox escalation and EMT drift rather than induce acute cytotoxicity. This strategy is particularly relevant in metabolically stressed hosts, such as patients with type 2 diabetes, where systemic oxidative pressure predisposes tumors toward premature redox adaptation [111].

### 4.2 Conditioning redox-intermediate tumors for therapeutic sensitization (state II–III)

Hybrid epithelial–mesenchymal states represent a critical therapeutic inflection point, characterized by emerging antioxidant reprogramming, partial ferroptosis resistance, and increasing immune modulation. In this redox-intermediate window, phytomedicine may exert its greatest clinical leverage as a conditioning agent that destabilizes adaptive redox circuitry prior to definitive therapy [112]. Mechanistically, multiple phytochemicals have been shown to interfere with NRF2 transcriptional persistence, cystine transport, lipid remodeling, and mitochondrial redox buffering, thereby lowering the ferroptosis threshold without fully abrogating cell viability [113]. Importantly, such partial redox destabilization can re-sensitize tumors to chemotherapy, radiotherapy, or immune checkpoint blockade by restoring lipid peroxidation–associated danger signaling and immunogenic cell death pathways [114]. Decision rules emerging from these studies suggest that phytomedicine should be introduced before or in early combination with standard therapies, rather than after overt resistance has consolidated. This temporal positioning distinguishes redox-conditioning from empiric add-on strategies and aligns phytomedicine with state-adaptive therapeutic logic.

### 4.3 Targeting redox-high/ferroptosis-resistant states (state III–IV)

In advanced redox-adapted tumors characterized by sustained NRF2 activation, reinforced GPX4/FSP1-dependent ferroptosis defense, and immune exclusion, phytomedicine alone is unlikely to induce tumor regression. However, evidence indicates that selected phytochemicals can selectively disrupt nodal vulnerabilities within the redox defense network, particularly when used in rational combinations [115]. For example, compounds targeting lipid metabolism, iron handling, or mitochondrial electron transport can compromise ferroptosis buffering indirectly, creating synthetic vulnerabilities exploitable by pharmacologic GPX4 inhibition or immune-based therapies [116]. In this setting, phytomedicine functions not as a primary cytotoxic agent but as a network perturbator, weakening the robustness of redox-locked tumor states and expanding the therapeutic window for otherwise ineffective interventions.

### 4.4 Decision rules for redox-guided integration of phytomedicine

Collectively, these findings support a redox-guided framework in which phytomedicine is deployed according to tumor state rather than uniformly across disease stages. In redox-low tumors, phytomedicine serves a stabilizing role; in intermediate states, it functions as a conditioning and sensitizing agent; and in redox-high states, it operates as a network-level disruptor within combination regimens [112,113,[116], [117], [118], [119], [120], [121], [122]]. Importantly, this framework reframes phytomedicine from an empirical alternative therapy into a state-aware component of precision oncology, aligned with dynamic tumor adaptation rather than static molecular classification.

## 5 Clinical stratification of redox-adapted cancers

Redox adaptation is increasingly recognized as a clinically actionable determinant of therapeutic response, rather than a purely mechanistic or experimental phenomenon. Accumulating clinical transcriptomic and outcome data indicate that tumors exhibiting coordinated activation of antioxidant defense programs, EMT-associated plasticity, and immune modulatory pathways constitute a distinct, high-risk clinical subgroup characterized by treatment refractoriness and early relapse [123].

Importantly, this high-risk subgroup is not defined by a single oncogenic mutation or pathway but instead reflects a convergent functional state that emerges under sustained therapeutic and microenvironmental stress, underscoring the limitations of genotype-only stratification paradigms.

### 5.1 Redox–cell death signatures as prognostic and predictive classifiers

Recent integrative analyses demonstrate that composite cell-death–related gene signatures, particularly those incorporating ferroptosis-associated genes, can robustly stratify patients according to prognosis, immune responsiveness, and drug sensitivity. For example, combined ferroptosis–cuproptosis signatures enriched for redox-regulatory genes such as G6PD, SRXN1, TXNRD1, and SQSTM1 identify tumors with elevated antioxidant buffering capacity, increased stemness indices, and immunosuppressive microenvironments [124]. These composite signatures capture a systems-level redox buffering phenotype that reflects the balance between oxidative stress generation and detoxification, rather than isolated expression of individual ferroptosis regulators.

Clinically, these high-risk tumors display inferior overall survival but paradoxically show differential sensitivity to specific targeted agents or immunotherapies, highlighting the value of redox-based stratification beyond conventional staging systems [125]. Importantly, such composite signatures outperform single-gene biomarkers by capturing functional redox states that integrate metabolic rewiring, oxidative stress tolerance, and cell-death resistance programs rather than isolated molecular alterations [126]. Such state-based classifiers provide a rational framework for identifying patients who may benefit from redox-modulating combination therapies, including ferroptosis sensitizers or metabolic–redox co-targeting strategies.

### 5.2 EMT-coupled redox adaptation and therapy resistance

Clinical datasets across multiple solid tumors consistently reveal that EMT-high or hybrid epithelial/mesenchymal states co-segregate with enhanced redox defense activation. Tumors enriched for EMT transcriptional programs frequently exhibit upregulation of the NRF2–SLC7A11–GPX4 axis, conferring resistance not only to apoptosis but also to ferroptosis-inducing therapies [127]. This EMT–redox coupling generates a dual-protective phenotype in which lineage plasticity and oxidative stress buffering reinforce one another, enabling survival under cytotoxic, targeted, and radiotherapeutic pressure. This coupling explains, at least in part, why EMT-associated tumors often persist after chemotherapy, targeted therapy, or radiotherapy despite apparent initial responses [128]. From a clinical stratification perspective, EMT status alone is insufficient; instead, integration of EMT features with redox-adaptive markers provides a more precise framework to identify patients at high risk for therapeutic failure and disease recurrence [129]. Accordingly, EMT–redox composite classifiers may serve as superior predictors of minimal residual disease and early relapse compared with EMT or redox markers assessed in isolation.

### 5.3 Extracellular Vesicle–Based biomarkers and systemic immune suppression

Beyond tumor-intrinsic features, redox-adapted cancers actively remodel systemic immunity through extracellular vesicle (EV)–mediated signaling. Seminal clinical studies have demonstrated that exosomal PD-L1 levels in patient plasma correlate with tumor burden, immune suppression, and responsiveness to immune checkpoint blockade [130]. Dynamic changes in circulating exosomal PD-L1 during therapy stratify responders from non-responders more effectively than tissue PD-L1 expression alone [131]. This discrepancy underscores the advantage of EV-based biomarkers in capturing treatment-induced immune modulation that static tissue biopsies fail to reflect.

Subsequent clinical and translational studies further revealed that EV-associated cargos—including redox-regulated microRNAs and immune-modulatory proteins—serve as minimally invasive liquid biopsy markers reflecting the functional redox and immune state of tumors in real time [132]. Collectively, these findings position EV profiling as a critical component of redox-based clinical stratification by integrating tumor-intrinsic redox adaptation with systemic immune consequences. Importantly, EV signatures provide a readout of tumor–host interaction rather than tumor biology alone.

### 5.4 Liquid biopsy and dynamic monitoring of redox states

Liquid biopsy technologies, encompassing circulating tumor cells (CTCs), circulating tumor DNA (ctDNA), and EV-derived nucleic acids, offer a powerful platform for longitudinal assessment of redox adaptation. Notably, EMT-like CTC subsets, which frequently evade EpCAM-based detection, are enriched for oxidative stress response and survival pathways, underscoring their relevance to therapy resistance and metastatic progression [133]. These mesenchymal or hybrid CTC populations represent a clinically underappreciated reservoir of redox-adapted cells capable of driving relapse despite apparent radiographic response.

Advances in circulating transcriptome analysis now enable detection of redox- and ferroptosis-related RNA signatures in blood, creating opportunities for real-time monitoring of tumor evolution under therapeutic pressure [134]. However, despite strong observational evidence, prospective interventional trials validating redox-guided treatment decisions remain limited, representing a critical translational gap [135]. Addressing this gap will require biomarker-integrated trial designs that prospectively assign therapy based on evolving redox states rather than baseline molecular features alone.

### 5.5 Toward a state-based oncology framework

Collectively, these findings support a shift from static, genotype-centric classification toward a state-based oncology framework, in which tumors are stratified according to functional redox adaptation states integrating ferroptosis resistance, EMT plasticity, and immune evasion mechanisms [136].

Such an approach enables identification of patients unlikely to benefit from standard regimens, guides rational combination strategies (e.g., redox modulation plus immunotherapy), and supports adaptive treatment adjustment based on dynamic biomarker readouts. In this framework, redox adaptation is redefined not as a terminal resistance endpoint but as a measurable, targetable, and potentially reversible tumor state, thereby transforming redox biology from a descriptive paradigm into an actionable clinical strategy.

### 5.6 Framework proposal: redox State–Matched phytomedicine as a decision-modulating strategy

Therapeutic failure in metabolically compromised cancers such as type 2 diabetes–associated pancreatic ductal adenocarcinoma (PDAC) increasingly reflects a mismatch between intervention logic and tumor redox state, rather than an absence of actionable targets. Building on the state-based redox–EMT framework outlined above, we propose a decision-modulating phytomedicine framework in which multi-component herbal formulations are deployed not as uniform cytotoxic agents, but as state-matched modulators of redox vulnerability [[137], [138], [139]].

At early or intermediate redox-adaptive states—characterized by partial EMT, inducible antioxidant responses, and preserved ferroptotic competence—phytomedicines enriched in redox-buffering or redox-sensitizing constituents may function to destabilize emerging antioxidant programs without provoking compensatory stress hardening. Experimental evidence indicates that phytochemical mixtures targeting mitochondrial ROS flux, NADPH homeostasis, and lipid peroxidation thresholds can shift tumors from an adaptive to a vulnerable redox window, thereby enhancing responsiveness to cytotoxic or immune-based therapies [140,141]. In this context, phytomedicine acts as a priming modality, modulating redox tone rather than enforcing direct tumor eradication.

In contrast, advanced redox-addicted states—marked by sustained NRF2 activation, reinforced ferroptosis defense (GPX4, SLC7A11, FSP1), and immune exclusion—require a fundamentally different intervention logic. Here, indiscriminate antioxidant supplementation or ROS induction may further entrench resistance. Instead, phytomedicines with context-dependent redox-disruptive capacity, particularly those capable of attenuating NRF2-centered transcriptional dominance or interfering with lipid remodeling and mitochondrial redox buffering, may serve as decision modifiers that re-open ferroptotic or immunogenic vulnerabilities [117,118,[142], [143], [144]].

Importantly, the therapeutic value of phytomedicine in this framework does not derive from single-compound potency, but from multi-node engagement across redox, metabolic, and stromal axes. Such multi-target coverage mirrors the systems-level nature of redox adaptation itself, which emerges from coordinated tumor–stroma–immune interactions rather than isolated oncogenic lesions. Preclinical models demonstrate that multi-component herbal formulations can simultaneously modulate intracellular antioxidant buffering, EMT plasticity, and extracellular vesicle–mediated immune suppression—features that are difficult to address with single-target agents alone [120,121,[145], [146], [147], [148]].

Crucially, this framework does not advocate phytomedicine as a replacement for standard anticancer therapies. Rather, it positions phytomedicine as a decision-modulating layer that informs when and how cytotoxic, targeted, or immune-based interventions should be deployed. By aligning phytomedicine selection with tumor redox state, clinicians may reduce futile treatment escalation, minimize redox-driven resistance amplification, and improve the durability of downstream therapeutic responses.

Collectively, this proposal reframes phytomedicine from an empiric adjunct into a state-aware therapeutic instrument, integrated within a redox-guided oncology paradigm. While prospective validation remains necessary, the convergence of mechanistic redox biology and emerging translational evidence supports the feasibility of redox state–matched phytomedicine as a rational proof-of-concept strategy in diabetes-associated and metabolically reprogrammed PDAC.

### 5.7 Redox State–Matched phytomedicine as a decision-modulating strategy

Cancer therapy failure increasingly reflects an inability to account for state-dependent redox adaptation rather than the absence of therapeutic agents. A growing body of experimental evidence indicates that multiple phytochemicals can modulate oxidative stress tolerance, ferroptosis sensitivity, or NRF2-centered antioxidant buffering; however, these effects are highly context-dependent and vary across tumor stages and redox states. Importantly, such observations do not support the notion that phytochemicals are universally beneficial, but instead underscore that their therapeutic impact is contingent upon underlying tumor pathology and redox configuration [97,149].

From a decision-oriented perspective, this implies that phytomedicine should not be conceptualized as a standalone or indiscriminate intervention. Rather, multi-component formulations and rational combinations may be required to engage distinct redox vulnerabilities that emerge during tumor progression. In this framework, the role of phytomedicine is not to replace conventional therapy, but to condition or reprogram redox-adapted tumor states that are otherwise refractory to single-agent approaches [139,144].

Within this conceptual space, multi-herbal formulations such as BK002 and SH003 can be positioned as illustrative examples rather than case studies. These formulations integrate multiple redox-active principles within a single platform, aligning with the notion that complex, state-dependent resistance cannot be effectively addressed through single-target strategies. They therefore serve as proof-of-concept prototypes demonstrating how redox state–matched phytomedicine may be operationalized within a broader decision-modulating therapeutic framework, rather than as universally applicable treatments [150,151].

## 6 Decision-oriented therapeutic strategies

The failure of uniform treatment paradigms in advanced cancer underscores the need for decision-oriented therapeutic frameworks that explicitly account for tumor redox states. Rather than treating oxidative stress as a static vulnerability, contemporary evidence supports a model in which cancers occupy distinct redox-adapted states, each governed by different survival logics and therefore requiring tailored intervention strategies. In this context, redox biology provides not only mechanistic insight but also a decision-relevant layer that informs when, how, and in whom specific therapies should be deployed.

### 6.1 Redox State–Driven therapeutic vulnerabilities

Tumors characterized by high antioxidant buffering capacity—typically marked by elevated NRF2 activity, SLC7A11 expression, and GPX4-dependent lipid peroxide detoxification—exhibit intrinsic resistance to therapies that rely on oxidative damage accumulation, including chemotherapy, radiotherapy, and certain immunotherapies [152]. In such contexts, further ROS induction alone may paradoxically reinforce adaptive stress responses rather than induce cell death [153]. This phenomenon reflects a threshold shift in oxidative tolerance, whereby redox-adapted tumors convert cytotoxic ROS signals into pro-survival transcriptional and metabolic programs.

Conversely, these tumors exhibit synthetic vulnerabilities to strategies that disable ferroptosis defense systems, such as GPX4 inhibition, cystine transport blockade, or disruption of CoQ-dependent redox buffering via FSP1-related mechanisms [154]. Importantly, accumulating evidence indicates that ferroptosis sensitivity is highly state-dependent and context-specific, shaped by metabolic flux, mitochondrial competence, and iron availability rather than by single pathway inhibition alone. Emerging studies further suggest that such interventions must be applied within defined redox windows, as excessive or poorly timed inhibition may trigger compensatory metabolic rerouting rather than effective tumor collapse [155]. This temporal dimension emphasizes redox adaptation as a dynamic variable, reinforcing the need for biomarker-guided timing rather than static drug selection.

### 6.2 Integrating redox modulation with immune-based therapies

Redox-adapted tumors frequently coexist with immune-excluded or immune-suppressed microenvironments, driven in part by EMT-associated immune evasion programs and redox-regulated expression of immune checkpoint molecules such as PD-L1 [156]. Notably, excessive antioxidant activity within tumor cells can dampen immunogenic cell death (ICD), thereby limiting antigen release and blunting T cell priming following cytotoxic therapy [157]. This establishes a mechanistic link between intracellular redox buffering and failure of immune activation, reframing immune resistance as a redox-conditioned phenotype rather than solely an immunologic defect.

Decision-oriented strategies therefore prioritize sequencing and combination logic, wherein redox-modulating interventions are deployed to re-sensitize tumors prior to or concurrently with immune checkpoint blockade. For example, transient disruption of ferroptosis defense or mitochondrial redox homeostasis may enhance lipid peroxidation–associated danger signals, restore dendritic cell activation, and improve responsiveness to PD-1/PD-L1 inhibitors [158]. Such approaches conceptualize redox modulation as an immune-permissive conditioning step that lowers the activation threshold for antitumor immunity rather than functioning as a direct cytotoxic modality [159].

### 6.3 Stratified combination therapy in metabolically compromised hosts

In metabolically complex patient populations—such as those with type 2 diabetes, obesity, or chronic inflammation—systemic redox balance introduces an additional layer of complexity to therapeutic decision-making. Clinical and preclinical studies indicate that metabolic modulators, including agents affecting insulin signaling, mitochondrial flux, or lipid metabolism, can indirectly reshape tumor redox states and alter therapeutic sensitivity [160]. These systemic influences are particularly relevant in cancers arising within chronically inflamed or hyperglycemic microenvironments, where redox adaptation may be established prior to therapeutic intervention [161].

Accordingly, stratified combination strategies that integrate metabolic control with redox-targeted therapy are increasingly recognized as clinically rational. Rather than intensifying cytotoxic pressure, these approaches aim to destabilize the tumor's redox–metabolic equilibrium, thereby lowering the threshold for ferroptosis or immune-mediated clearance [162]. This strategy aligns with a systems-level view of cancer vulnerability, in which redox imbalance emerges from coordinated metabolic, immune, and stromal interactions rather than tumor-intrinsic alterations alone.

### 6.4 Toward redox-guided clinical decision algorithms

Collectively, these insights support a transition from empiric combination therapy toward redox-guided clinical decision algorithms, in which therapeutic choices are informed by functional biomarkers rather than tumor type alone. Candidate biomarkers include lipid peroxidation signatures, GPX4/FSP1 expression profiles, NRF2 transcriptional activity, EMT status, and exosomal cargo reflecting systemic redox adaptation [163]. Crucially, these markers capture dynamic tumor states rather than static genetic lesions, enabling adaptive treatment strategies responsive to tumor evolution under therapeutic pressure.

Such frameworks align with the broader movement toward state-based oncology, wherein treatment is tailored to evolving tumor phenotypes rather than fixed genetic alterations. By matching therapeutic logic to redox state, clinicians may more effectively overcome therapy resistance, reduce unnecessary toxicity, and unlock durable responses in otherwise refractory cancers [164].

## 7 Translational roadmap and clinical trial design

**Fig. 2:**
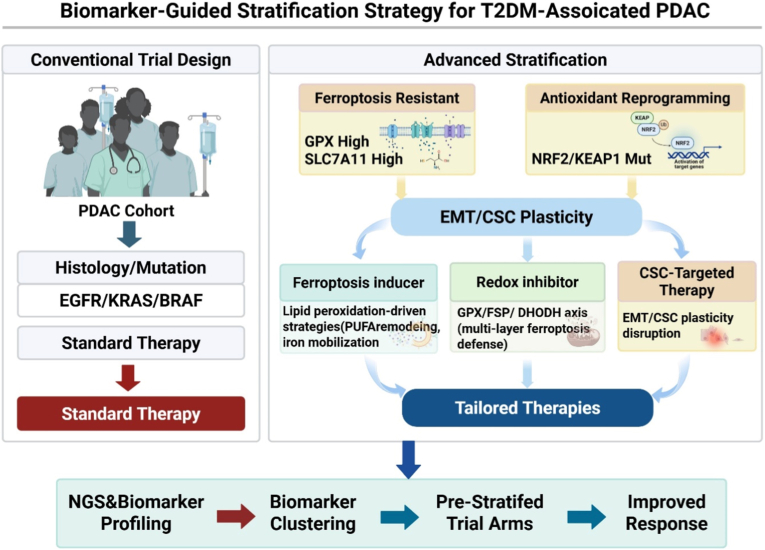
Biomarker-Guided Stratification Strategy for T2DM-Associated PDAC. Schematic comparison between conventional trial designs based on histopathology and single-gene alterations and an advanced stratification framework incorporating ferroptosis resistance, antioxidant reprogramming, and EMT/CSC plasticity. The proposed approach enables pre-stratified therapeutic allocation using tissue- and liquid biopsy–derived redox biomarkers, facilitating personalized and mechanism-informed clinical decision-making in PDAC. Abbreviations: Cancer stem cell (CSC); Epithelial–mesenchymal transition (EMT); Glutathione peroxidase 4 (GPX4); Kelch-like ECH-associated protein 1 (KEAP1); Nuclear factor erythroid 2–related factor 2 (NRF2); Pancreatic ductal adenocarcinoma (PDAC); Solute carrier family 7 member 11 (SLC7A11); Type 2 diabetes mellitus (T2DM); Zinc finger E-box-binding homeobox 1 (ZEB1).

Accumulating clinical evidence indicates that therapeutic failure in advanced cancers is driven less by the absence of druggable targets than by inadequate patient stratification that fails to account for tumor redox adaptation states. Conventional trial designs largely rely on histopathologic classification or single genomic alterations; however, these approaches do not sufficiently capture dynamic phenotypic programs such as EMT-associated redox reprogramming and ferroptosis resistance, which critically shape treatment responsiveness [165]. Building on state assignment, we propose a biomarker-guided stratification workflow that pre-allocates patients to ferroptosis/redox/CSC-informed trial arms rather than relying solely on histology or single-gene alterations (Fig. 2). This structure explicitly links NRF2/KEAP1 status and ferroptosis-defense markers (e.g., GPX4, SLC7A11) to tailored combination strategies.

### 7.1 Biomarker-guided patient stratification

As summarized in Table 1, redox adaptation can be operationally classified into discrete tumor states distributed along the EMT–redox spectrum. State I reflect an epithelial-dominant phenotype with low EMT plasticity and minimal antioxidant reprogramming, in which ferroptosis as a regulated cell death program remains largely intact due to limited NRF2/GPX4 buffering capacity [166,167]. State II represents an intermediate or hybrid epithelial–mesenchymal state, where partial EMT features coexist with emerging redox-adaptive programs, conferring increased tolerance to oxidative stress and early resistance to ferroptosis as well as conventional cytotoxic therapies [48,168].

**Table 1:** Redox–EMT–ferroptosis–metabolic state–guided clinical stratification framework.

Tumor Redox–EMT State	Key Molecular Features	Cellular/Stromal Context	Host Metabolic Context	Clinical Readout/Feasible Biomarker	Representative Clinical Scenario	Predicted Therapy Resistance	Typical Clinical Scenario	Rational Therapeutic Strategy	Refs
State I: Redox-low/EMT-low	Low NRF2 activity, intact lipid peroxidation, GPX4-low	Epithelial tumor cells, minimal CAF/PSC activation	Metabolically normal	Low NRF2 score, GPX4-low IHC	Treatment-naïve PDAC	Chemotherapy, radiotherapy sensitivity preserved		Standard cytotoxic therapy ± immunotherapy	[166,167]
State II: Intermediate redox/Hybrid EMT	Partial EMT, moderate NRF2–SLC7A11 activation	Emerging CSC-like cells, early CAF support	Mild metabolic stress	Mixed EMT markers, moderate SLC7A11	Suboptimal response to first-line chemo	Adaptive resistance, transient responses	State II: post-chemotherapy minimal residual disease	Redox-sensitizing agents + chemotherapy	[48,168]
State III: Redox-high/EMT-high CSC-enriched	High NRF2, GPX4, SLC7A11; ferroptosis resistance	EMT-high CSCs, active PSC/CAF paracrine signaling	Type 2 diabetes, hyperinsulinemia, chronic inflammation	High NRF2 score, GPX4/SLC7A11 IHC, HbA1c↑, fasting insulin↑	Post-FOLFIRINOX relapse in T2DM PDAC	Resistance to KRAS-targeted therapy, chemo, ICI	State III: post-FOLFIRINOX relapse	Ferroptosis defense disruption (GPX4/FSP1) + redox modulation	[169,170]
State IV: Redox-high/Immune-excluded	NRF2-driven antioxidant dominance, exosomal PD-L1	CAF-rich stroma, immune suppression	Metabolic syndrome	High circulating exosomal PD-L1, low CD8 infiltration	ICI non-responder with fibrotic stroma	Immunotherapy failure	State IV: ICI non-responder with dense stroma	Redox disruption → immune priming → ICI	[171,172]
State V: Dynamic redox-adaptive relapse state	Temporal NRF2/ferroptosis reprogramming	Therapy-selected EMT-CSC niches	Variable	Rising ferroptosis-resistance RNA in liquid biopsy	Early KRAS inhibitor resistance (e.g., G12C)	Rapid relapse after initial response	State V: late-stage T2DM PDAC with cachexia	Adaptive, biomarker-guided combination therapy	[173,174]

Abbreviations: ↑, upregulation; ↓, downregulation. Cancer-associated fibroblast (CAF); coenzyme Q10 **(**CoQ10**)**; cancer stem cell **(**CSC**)**; circulating tumor cell (CTC); circulating tumor DNA **(**ctDNA**)**; dihydroorotate dehydrogenase **(**DHODH); epithelial–mesenchymal transition; EV, extracellular vesicle (EMT); ferroptosis suppressor protein 1 (FSP1); glutathione peroxidase 4 (GPX4); glycated hemoglobin (HbA1c); immune checkpoint inhibitor (ICI); insulin-like growth factor-1 receptor (IGF-1R); interleukin-6 (IL-6); Kirsten rat sarcoma viral oncogene homolog (KRAS); mesenchymal stem cell (MSC); nuclear factor erythroid 2–related factor 2 (NRF2); pancreatic ductal adenocarcinoma (PDAC); programmed death-ligand 1(PD-L1); pancreatic stellate cell (PSC); reactive oxygen species (ROS); cystine/glutamate antiporter light chain (SLC7A11); type 2 diabetes mellitus (T2DM); transforming growth factor-beta (TGF-β).

Progression to State III is defined by stabilization of redox-adaptive circuitry, characterized by sustained NRF2 activation, reinforced ferroptosis defense through GPX4, SLC7A11, and FSP1, and pronounced EMT-associated plasticity. Clinically, this state frequently coincides with post-treatment persistence, stromal reinforcement, and the onset of immune suppression, marking a critical inflection point at which redox adaptation transitions from inducible to functionally entrenched [169,170].

At the extreme end, State IV denotes a redox-addicted tumor ecosystem in which tumor cells, stromal compartments, and extracellular vesicle–mediated signaling cooperatively stabilize ferroptosis resistance, immune evasion, and metabolic resilience, rendering single-modality or pathway-selective interventions largely ineffective [171,172].

Importantly, State V represents a terminal redox-locked condition in which adaptive programs become constitutive and self-sustaining rather than stress-responsive. Tumors in this state exhibit fixed EMT/stem-like identity, extreme antioxidant dependency, dense stromal insulation, and systemic immune exclusion, collectively defining a boundary beyond which current redox- or ferroptosis-targeted strategies are unlikely to yield durable benefit. In this context, prevention of progression into State V—rather than therapeutic reversal—emerges as the dominant clinical objective [173,174].

Redox-adapted tumors exhibit reproducible molecular signatures that can be leveraged for clinical stratification. These include elevated expression of ferroptosis defense markers (GPX4, SLC7A11, FSP1), sustained activation of NRF2-dependent antioxidant transcriptional programs, and EMT-associated adhesion remodeling markers such as reduced E-cadherin and increased N-cadherin or vimentin [175]. Importantly, these features frequently coexist within hybrid epithelial–mesenchymal states rather than fully mesenchymal phenotypes, underscoring the need for composite biomarker panels rather than single-marker selection strategies [176]. Recent translational studies demonstrate that patients whose tumors exhibit high ferroptosis resistance signatures show inferior responses to both cytotoxic chemotherapy and immune checkpoint blockade, suggesting that redox adaptation constitutes an independent resistance layer beyond canonical oncogenic drivers [177]. Accordingly, incorporation of redox-state biomarkers into eligibility criteria or stratification arms may improve predictive accuracy and reduce trial attrition due to biologically mismatched patient populations.

### 7.2 Adaptive and enrichment trial designs

Given the dynamic and reversible nature of redox adaptation, adaptive clinical trial designs represent a particularly suitable framework for testing redox-targeted interventions. Adaptive enrichment strategies allow real-time modification of inclusion criteria based on interim biomarker analyses, enabling selective enrichment of patients exhibiting targetable redox vulnerabilities while discontinuing exposure in non-responsive subgroups [178]. Such designs have been increasingly endorsed by regulatory agencies to enhance efficiency, minimize patient risk, and improve translational relevance [179].

In the context of redox-based therapies, adaptive designs may integrate serial biomarker monitoring—including circulating redox markers, exosomal cargo profiling, or transcriptional redox signatures—to dynamically refine patient selection and dosing strategies. This approach directly addresses a major limitation of prior antioxidant or ferroptosis-inducing trials that failed to confirm on-target redox modulation in vivo [180].

### 7.3 Decision rules linking redox state to therapeutic modality

A clinically actionable framework emerges when redox adaptation states are explicitly linked to therapeutic logic. Tumors characterized by high ferroptosis defense and NRF2 activation may benefit from strategies that directly disrupt antioxidant buffering systems or exploit synthetic lethality through combined ferroptosis induction and adhesion pathway interference [181]. Conversely, tumors with intermediate redox adaptation may be more amenable to redox-sensitizing approaches combined with immunotherapy, particularly when exosomal immune suppressive signals such as PD-L1 are prominent [182]. Embedding these decision rules into trial protocols enables rational treatment matching rather than empirical escalation, shifting trial objectives from drug-centric efficacy testing to state-centric therapeutic validation [183].

### 7.4 Translational outlook

Ultimately, biomarker-guided and adaptive trial designs tailored to redox adaptation represent a critical step toward overcoming recurrent failures in oncology drug development. By integrating mechanistic redox biology with clinically actionable stratification strategies, future trials may more effectively identify responsive patient subsets, accelerate proof-of-concept validation, and translate redox-targeted interventions from experimental promise to durable clinical benefit [184].

## 8 Limitations and future directions

Despite growing recognition of redox adaptation as a central determinant of therapeutic resistance, several limitations currently constrain its clinical translation. First, redox adaptation represents a dynamic and reversible tumor state rather than a static molecular attribute. As a result, single time-point biopsies or baseline genomic profiling may fail to capture therapy-induced shifts in antioxidant buffering, EMT plasticity, or ferroptosis susceptibility under treatment pressure [178,179].

Second, although composite redox- and EMT-associated gene signatures demonstrate strong prognostic and predictive value, their analytical standardization remains limited. Variability in platform selection, cutoff thresholds, tumor purity, and stromal admixture complicates cross-cohort validation and clinical implementation [58,185].

Third, many redox-targeted strategies have advanced without rigorous pharmacodynamic confirmation of on-target redox modulation in patient tumors [181]. Emerging evidence suggests that failure to verify redox-state modulation, rather than target irrelevance, underlies many negative clinical outcomes [182].

Fourth, redox adaptation is inseparable from host metabolic status, immune contexture, and stromal remodeling. Clinical datasets indicate that conditions such as type 2 diabetes, obesity, and chronic inflammation precondition tumors toward antioxidant dominance and ferroptosis resistance, yet these variables are rarely incorporated into trial stratification [160,161].

Finally, although extracellular vesicle–based biomarkers offer promise for dynamic redox monitoring, their integration into interventional trial designs remains limited [132,183]. Collectively, these limitations delineate clear future directions: (i) longitudinal biomarker strategies, (ii) standardized redox-state scoring systems, (iii) pharmacodynamic validation of redox modulation, and (iv) integration of host metabolic and immune contexts into trial design.

## 9 Conclusions: take-home clinical rules for redox-adapted cancers

Therapeutic resistance in advanced cancer should no longer be interpreted as a failure to identify druggable targets, but as a failure to recognize and therapeutically address adaptive tumor states.

Across solid malignancies, redox adaptation emerges as a unifying and clinically actionable framework integrating ferroptosis resistance, EMT-driven plasticity, metabolic rewiring, immune evasion, and extracellular vesicle–mediated communication.

Based on the evidence synthesized in this review, we propose a set of decision-oriented clinical rules that link tumor redox–EMT states to therapeutic strategy selection (Table 2).

**Table 2:** Clinical decision rules based on redox states.

Clinical Decision Rules Based on Redox States
**Rule 1**: If NRF2/GPX4-high tumors exhibit a hybrid EMT signature, ferroptosis resistance should be presumed even in the absence of overt therapy failure.
**Rule 2**: In T2DM-associated PDAC, post-FOLFIRINOX relapse should prompt redox-state assessment prior to empiric regimen escalation.
**Rule 3**: KRAS inhibitor failure accompanied by EMT transition suggests prioritizing redox/ferroptosis co-targeting over sequential KRAS inhibition.
**Rule 4**: Dynamic changes in exosomal PD-L1 may precede radiologic progression in redox-adapted tumors, supporting its use as an early warning biomarker.

First, redox adaptation constitutes a functional tumor state requiring integrative assessment of antioxidant defenses, EMT status, and immune context rather than reliance on single biomarkers.

Second, resistance to cytotoxic, targeted, and immune-based therapies frequently reflects elevated ferroptosis thresholds and NRF2-centered redox buffering within EMT-high and stem-like compartments.

Third, host metabolic conditions—including diabetes and chronic inflammatory states—actively shape tumor redox adaptation and should be incorporated into both patient stratification and therapeutic planning.

Importantly, effective targeting of redox-adapted cancers will require abandoning uniform treatment escalation strategies in favor of decision-oriented therapeutic logic that explicitly matches intervention modality to tumor redox state. Biomarker-guided stratification, adaptive enrichment trial designs, and rational combination strategies that disrupt multiple layers of redox defense represent essential components of this approach. In this context, redox modulation should be viewed not solely as a cytotoxic strategy, but as a conditioning mechanism capable of re-sensitizing tumors to immunotherapy and targeted agents. As redox biology transitions from experimental insight to clinical application, the principal challenge lies not in identifying additional targets but in deploying existing knowledge with clinical discipline, mechanistic clarity, and state-aware trial design. Integrating redox-state assessment into precision oncology frameworks offers a tangible path toward overcoming persistent therapeutic failure and achieving durable responses in otherwise refractory malignancies.

## Funding

This work was supported by the Basic Science Research Program through the National Research Foundation of Korea (NRF) funded by the Ministry of Education (RS-2020-NR054734); the 10.13039/501100003725National Research Foundation of Korea (NRF) grant funded by the Korea government (10.13039/501100014188MSIT) (RS-2020-NR049559, RS-2024-00350362); the Korea 10.13039/100018696Health Technology R&D Project through the 10.13039/501100003710Korea Health Industry Development Institute (10.13039/501100003710KHIDI)**,** funded by the 10.13039/100009647Ministry of Health & Welfare, Republic of Korea (RS-2020-KH087790); and the TIPS Program (No. RS-2024-00507224) funded by the 10.13039/501100013129Ministry of SMEs and Startups (MSS, Korea).

## CRediT authorship contribution statement

**Moon Nyeo Park:** Conceptualization, Data curation, Funding acquisition, Investigation, Resources, Supervision, Validation, Writing – original draft, Writing – review & editing. **Hyo Jeong Kim:** Formal analysis, Software, Visualization, Writing – original draft, Writing – review & editing. **Sohyun Park:** Methodology, Writing – original draft. **Rony Abdi Syahputra:** Software, Writing – original draft, Writing – review & editing. **Domenico V. Delfino:** Writing – original draft, Writing – review & editing. **Seong-Gyu Ko:** Project administration, Writing – original draft, Writing – review & editing. **Bonglee Kim:** Project administration, Writing – original draft, Writing – review & editing.

## Declaration of competing interest

The authors declare that they have no known competing financial interests or personal relationships that could have appeared to influence the work reported in this paper.

This review was conducted independently and did not receive any specific grant from funding agencies in the public, commercial, or not-for-profit sectors. All authors have read and approved the final manuscript and declare no conflict of interest related to this submission.

## Data Availability

No data was used for the research described in the article.
